# Analysis of Medical Management in Geriatric Patients in the Hospital Emergency Department by Example of Selected Cities with County Status in Poland: A Retrospective Cohort Study

**DOI:** 10.3390/ijerph19010048

**Published:** 2021-12-21

**Authors:** Mariusz Celiński, Mateusz Cybulski, Joanna Fiłon, Marta Muszalik, Mariusz Goniewicz, Elżbieta Krajewska-Kułak, Anna Ślifirczyk

**Affiliations:** 1 Department of Emergency Medicine, Faculty of Health Sciences, Pope John Paul II State School of Higher Education in Biała Podlaska, 21-500 Biała Podlaska, Poland; manieek@poczta.onet.pl (M.C.); aslifirczyk1@gmail.com (A.Ś.); 2 Department of Integrated Medical Care, Faculty of Health Sciences, Medical University of Białystok, 15-096 Białystok, Poland; joanna.filon@umb.edu.pl (J.F.); elzbieta.krajewska-kulak@umb.edu.pl (E.K.-K.); 3 Department of Geriatrics, Faculty of Health Sciences, Collegium Medicum in Bydgoszcz, University of Nicolaus Copernicus in Toruń, 85-094 Bydgoszcz, Poland; muszalik@cm.umk.pl; 4 Interfaculty Centre for Didactics, Department of Emergency Medicine, Medical University of Lublin, 20-093 Lublin, Poland; mariusz.goniewicz@gmail.com

**Keywords:** hospital emergency departments, elderly, emergency medicine, older adults, seniors, geriatrics, cardiovascular diseases, respiratory diseases, injuries

## Abstract

The aim of this study was to analyse medical management in geriatric patients in the Hospital Emergency Departments in the Biała Podlaska County and Chełm County (Poland) between 2016 and 2018 in a group of patients ≥65 years of age. We analysed medical records of 829 patients transported to Hospital Emergency Departments by Medical Emergency Teams. The research was conducted in the period from June 2019 to March 2020. We analysed emergency medical procedure forms and medical records of patients transported to the hospitals. Cardiovascular diseases were diagnosed in 40% of patients. Mortality cases accounted for 3.1% of the 1200 interventions analysed. Ambulance dispatch resulted in the patient being transported to the Hospital Emergency Departments in more than ^2^/_3_ of cases. The concordance between the diagnoses made by the Medical Emergency Teams and those made at the Hospital Emergency Departments was confirmed for 78% patients admitted to the department (n = 647), whereas the concordance of classification at the group level was estimated at 71.7% (n = 594). Further in-patient treatment was initiated in some of the patients admitted to the department (n = 385). The mean time of hospital stay was 10.1 days. In conclusion, differences between the initial diagnosis made by the heads of the Medical Emergency Teams and the diagnosis made by the doctor on duty in the Hospital Emergency Departments depended on the chapter of diseases in the ICD-10 classification, but they were acceptable. The majority of the patients were transported to Hospital Emergency Departments. The most common groups of diseases that require Hospital Emergency Departments admission include cardiovascular diseases, injuries due to external causes, and respiratory diseases. A moderate percentage of patients were qualified for further specialist treatment in hospital departments.

## 1. Introduction

Population ageing, manifested by an inevitable increase in the share of older people in the population structure, is a progressive demographic phenomenon observed in all countries worldwide. According to UN data, there were 727 million persons aged ≥65 years worldwide in 2020. This number is expected to more than double to 1.5 billion in 2050 [1]. Furthermore, it is expected that the percentage of individuals >80 years of age will increase significantly, from 137 million in 2017 to 425 million in 2050 [2]. The demographic forecast for Poland also raises concerns. It is estimated that the percentage of Poles ≥65 years of age will be about 25% in 2035, and that by 2060, Poland will be one of the oldest European communities [3].

The progressive global ageing of the population, particularly pronounced in Europe, gives rise to many threats, especially in the context of the health of older people [4]. In particular, it has a large and disproportionate impact on the functioning of Hospital Emergency Departments (HED). This trend is expected to increase [5,6]. The challenges that impact such a situation in the population of geriatric patients (≥65 years of age) primarily include multiple morbidities, atypical symptoms, polypharmacy and drug interactions, as well as misuse of prescription and over-the-counter medications [7,8]. Furthermore, older adults may present with functional disabilities, impaired cognition, and communication problems [9,10]. A significant percentage of the elderly also live alone [11,12,13,14,15]. For these reasons, visits of seniors to a HED should be treated as high-risk events [16,17].

Hospitalization is an important resource in the care of the older adults, and should be the final stage of therapy. Hospitalizations, especially if they are repeated and prolonged, can have negative health consequences for older patients, such as reduced functional performance, reduced quality of life, and increased frailty [18,19].

The main aim of this study was to assess geriatric patient management in the HED in Biała Podlaska and Chełm counties between 2016 and 2018 in a group of patients ≥65 years of age. Our goal was to analyse the procedures performed on the patient and the diagnostic process, and, thus, the final diagnosis made by a specialist doctor, as well as to compare the previous diagnosis made by the head of the Medical Emergency Team (MET) with the one made by the doctor in the HED. To this end, we analysed actions taken by medical personnel in provincial hospitals in Biała Podlaska and Chełm.

The following research questions were formulated:

1. What is the percentage of MET treated patients requiring admission to the HED?
2. What are the most common groups of diseases requiring admission to the HED?
3. How many patients transported to the HED require hospitalization in specialist hospital units?

## 2. Materials and Methods

### 2.1. Research Material

We analysed medical records of 1200 older patients (≥65 years) treated by METs from Biała Podlaska (600 sheets of medical records) and Chełm (600 sheets of medical records) in the Lublin Province (Poland). Although the emergency request forms were selected randomly, age ≥ 65 years was the main inclusion criterion. We analysed 2016–2018 data. We analysed 400 sheets of medical records in a year (200 sheets from a given county). The analyses covered 12 consecutive months of the year, which gave about 17 forms per month. 

**Figure 1:**
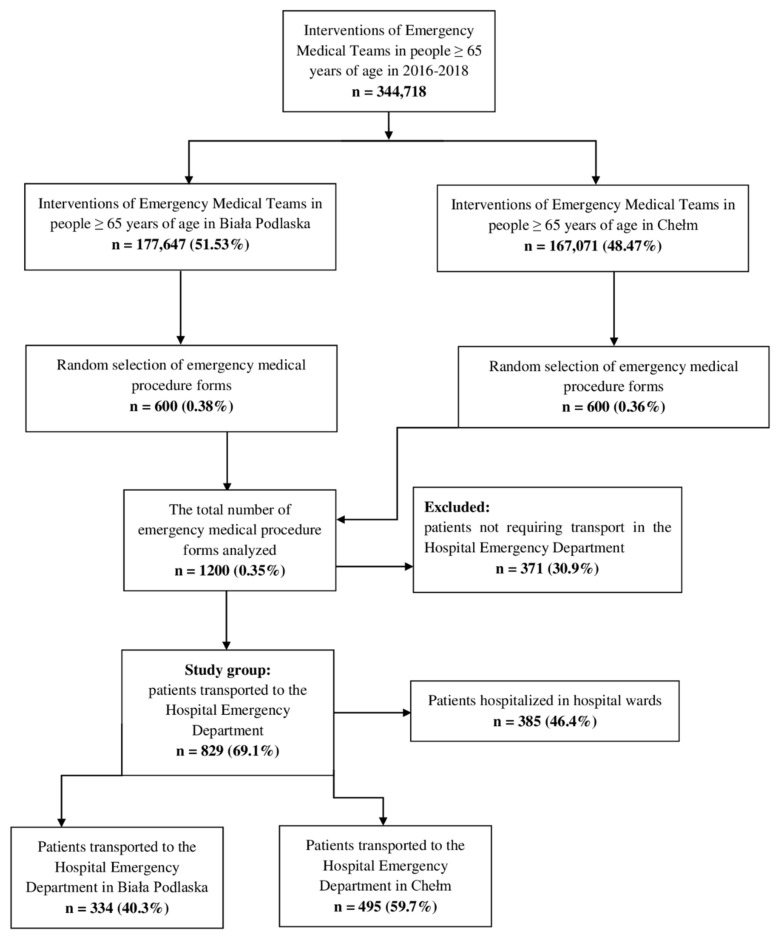
Patient enrolment in the study (a CONSORT diagram).

Based on the analysis of medical documentation, a group of 829 patients transported to HED by METs was identified. Following appropriate examination, these patients were qualified by the heads of the METs for transportation to the HED for further diagnosis and, if needed, further specialist in-patient treatment. Figure 1 shows patient enrolment in the study.

### 2.2. Research Methods

The research was conducted in the period from June 2019 to March 2020 at the Emergency Medical Service Station in Biała Podlaska (Biała Podlaska County) and the Medical Rescue Station in Chełm (Chełm County). Data collection was based on the analysis of emergency medical procedure forms, which are stored in the Emergency Medical Service Station in Biała Podlaska (Biała Podlaska County) and the Medical Rescue Station in Chełm (Chełm County). The obligation to use these forms is regulated by the Regulation of the Minister of Health on the types and scope of medical documentation and the manner in which it is processed, dated 21 December 2010. The emergency medical procedure form may be electronic or paper; however, it is always issued in two copies, one of which is given to the patient or their legal representative, and, in the case of transporting the patient to the hospital, it is handed in to the doctor on duty in the Hospital Emergency Department on a given day. Therefore, it is a medical documentation for the patient (if not transported to the hospital) or for HED personnel, and may be used as evidence in prosecutor’s proceedings; therefore, it must be carefully completed in each case [20]. We analysed the following data from the emergency medical procedure forms: diagnosis made by METs, and the decisions of the hospitals regarding admission or refusal to admit to the HEDs. 

We additionally analysed medical records of patients transported to the HED in the Specialist Hospital in Biała Podlaska and the Independent Public Provincial Specialist Hospital in Chełm, following previous management by medical personnel. This analysis was necessary to verify further patient management, and to compare the diagnosis made based on broader, in-patient diagnostic work-up. We analysed medical files of patients transported by METs to HEDs in Biała Podlaska and Chełm. We assessed patient management in HED, including medical procedures and treatment implemented in an older patient during their hospital stay. Finally, we compared the final diagnosis made by the doctor on duty in the emergency department with the one made by the head of the MET. We also evaluated the length of hospital stay in patients referred to a specialist department, and optional discharge for further outpatient treatment after diagnosis at HED.

In order to be able to achieve the set goals, we obtained prior consents for conducting the study from the heads of emergency medical institutions.

After submission of the application, and receiving approval from the Bioethics Committee of the Medical University of Bialystok (Approval No. R-I-002/26/2019 of 31 January 2019), we analysed medical documents.

### 2.3. Statistical Analysis

In the descriptive part, the characteristics of the studied population were recorded in the form of tables containing the percentage distribution of selected features or the values of selected descriptive statistics for numerical features. The following numerical characteristics of the parameters studied were most often estimated: arithmetic mean, median (middle value), the maximum and the minimum value, standard deviation (SD), as well as upper (c_75_) and lower (c_25_) quartile.

The verification of more complex research hypotheses required an analysis of the correlations between various features, and the selection of a statistical method depended on the nature of the compared parameters.

If both features were nominal (text), we compared the percentage distribution of variants of one feature in the compared groups, and assessed the significance of differences between them using the Chi-squared test of independence.

The analysis of the relationship between a nominal feature (e.g., the diagnosis made by the MET) and a numerical variable (e.g., patient’s age) consisted of comparing the values of descriptive statistics of the numerical feature in the compared groups. The significance of differences in the distribution of a numerical feature between the compared groups was assessed using the Mann–Whitney U test for two groups, and the Kruskal–Wallis test for three or more groups.

The data were supplemented with the results of the significance test of the correlation coefficient (*p*), which made it possible to assess whether the relationship found in the sample reflected a more general relationship in the entire population, or whether it was only incidental.

The level of statistical significance was set at *p* < 0.05, where:

- for *p* ≥ 0.05, there is no reason to reject the null hypothesis, which means that the tested difference, relationship, or effect is not statistically significant;
- for *p* < 0.05, there is a statistically significant relationship (*);
- *p* < 0.01 indicates a highly statistically significant relationship (**);
- *p* < 0.001 indicates an extremely highly statistically significant relationship (***).

## 3. Results

### 3.1. Socio-Demographic Characteristics of Patients 

The socio-demographic characteristics of patients whose medical records were analysed is shown in Table 1. Women accounted for the majority of the study population (almost 60% of all analysed cases of emergency interventions). The number of urban and rural patients was almost identical, which was not due to the deliberate selection of the sample. People with vocational education dominated in the study group of patients using emergency services, accounting for almost 50% of all patients. Although the study population included older adults, almost half of them were still married. Slightly less than half (42%) were widowed. Single and divorced patients accounted for 10% of the study group (Table 1).

The mean age of patients managed by METs was 77 years (SD of about 8 years). One in four patients was ≤70 years and ≥84 years of age. After a subdivision into 5-year age groups, patients aged 65–69 years and 80–84 years dominated. Female patients were older by an average of about 3.5 years (the difference was 6 years for comparison of median values). 

### 3.2. Diagnosis Made by METs

This section presents information on the diagnosis made by the head of the MET after examining the patient. The diagnosis was made according to the International Statistical Classification of Diseases and Related Health Problems (ICD-10), with the possibility of more than one diagnosis in the same patient; however, this was rare. This resulted from the fact that older patients may present with comorbidities affecting their current medical condition.

Due to the very large variety of classifications of patients, with more than 200 ICD-10 categories found in the study group of 1200 patients, the results were presented in the form of a simplified classification, i.e., by chapters and certain groups of diseases. 

The diagnoses classified based on ICD–10 chapters are presented in the table below (beginning with the most common ones). Cardiovascular diseases were diagnosed in 40% of patients, and the symptoms were not precisely classified in almost the same percentage of patients. Other diseases were less common, with the most common ones including injuries, poisoning, and other external factors, which were reported in one in eight patients (12.6%) (Table 2). Mortality cases accounted for 3.1% (1 in 30 patients) of the 1200 interventions analysed.

Classification into disease groups was shown for the most common chapters of ICD-10 (chapters diagnosed in at least 30 patients, i.e., IX, XVIII, XIX, X, IV, and V). 

“Other heart disease” accounted for almost half of the diagnoses in chapter IX, with arterial hypertension diagnosed in more than one in three patients, and cerebrovascular disease diagnosed in one in eight patients. Ischaemic heart disease was quite common (diagnosed in 28 patients). In Chapter XVIII, the most common diagnoses were not specified at the group level (general symptoms and signs in about 15%). These diagnoses accounted for about 38% of all Chapter XVIII diagnoses. The remaining diagnoses were more precise, with the most common including symptoms and signs involving the circulatory and respiratory systems (about 30% of Chapter XVIII diagnoses), as well as symptoms and signs involving the digestive system and abdomen (about 19%). Head injuries accounted for almost ^1^/_3_ of Chapter XIX diagnoses, with hip and thigh injuries in one in four cases, and with injuries to the trunk, upper limbs, and lower limbs accounting for 7–8% of cases. However, the percentage of individual groups of diseases did not exceed 5% in the entire population, with head injuries diagnosed in 4.3% of all MET-treated patients included in the analysis. Almost ^2^/_3_ of Chapter X diagnoses were chronic diseases of the lower respiratory tract, whereas influenza or pneumonia accounted for one in eight diagnoses in this chapter. Other clinical entities occurred incidentally. Diabetes mellitus accounted for almost of ¾ of Chapter IV diagnoses. Furthermore, the table also differentiates other glycaemic and metabolic disorders directly related to diabetes. Neurotic, stress-related, and somatoform disorders, as well as mood and mental disorders that had a direct impact on the functioning of a geriatric patient accounted for one in three Chapter V diagnoses (Table 3).

### 3.3. Further Patient Management by MET

This section discusses further MET management, i.e., transporting the patient to HED, following a thorough examination and an appropriate diagnosis. As can be seen in the table below, ^2^/_3_ of ambulance trips resulted in patient transportation to HED in a specialist hospital, where detailed diagnosis was initiated.

HED admissions were more common among men than women (72% vs. 67%); however, the difference was relatively small and was not statistically significant (the *p*-value was low, but technically above 0.05). It may be therefore concluded that there was no relationship between patient’s sex and the risk of the need for admission to HED.

However, there was a significant correlation between the need for admission to HED and patient’s age (*p* = 0.0034 **). Surprisingly, the correlation was found only for patients ≥90 years of age, who were less frequently transported to HED than their younger counterparts.

### 3.4. ICD–10 Classification of Diagnoses Made by an HED Doctor

There were some differences compared to the diagnosis made during MET intervention, with significantly less Chapter XVIII diseases, as shown in Table 4.

Classification into disease groups was shown for the most common chapters of ICD-10 classification (chapters diagnosed in at least 30 patients, i.e., IX, XIX, XVIII, X, and VI). The percentage was calculated for all 829 patients transported to HED and those diagnosed with a clinical entity in a given chapter. 

Chapter IX diagnoses mostly included heart diseases. A small percentage of the patients were also diagnosed with cerebrovascular diseases, hypertension, and ischaemic heart disease. Although Chapter XIX diagnoses varied significantly, injuries to the head, thigh, and hip accounted for nearly 60%. Injuries to the shoulder and upper arm accounted for one in ten Chapter XIX diagnoses. In Chapter XVIII, the most common diagnoses were unspecified symptoms involving the circulatory and respiratory systems (more than 40% of Chapter XVIII diseases), general symptoms and signs (26%), as well as symptoms and signs involving the digestive system and abdomen (22%). Most of Chapter X diagnoses were chronic diseases of the lower respiratory tract, and influenza or pneumonia (accounting for about ^1^/_3_ of respiratory diseases each). The vast majority of patients admitted to the HED due to diseases of the nervous system were classified as having episodic and paroxysmal disorders (more than 80% of Chapter VI diagnoses) (Table 5).

### 3.5. Diagnostic Compatibility between MET and HED

We verified whether there was a concordance between the general ICD-10 classification performed by METs and HED at the chapter and group level. The analysis included only 829 patients transported to HED.

The concordance at the level of disease chapters was confirmed for 78% of patients transported to HED (n = 647), whereas concordance at the level of group was estimated at 71.7% (n = 594). The diagnosis by an HED physician was considered consistent with MET diagnosis if any of the diagnoses made by MET (there were cases of two or three MET diagnoses, as three diagnoses for one patient may be reported in the emergency medical procedure form) was consistent with the diagnosis made by the emergency department physician (which was unambiguous).

Table 6 presents the percentage of patients transported to hospital HED in relation to the type of MET diagnosis by ICD-10 chapters. The table summarises data on the number of diagnoses in a given chapter made by MET, and the number of patients with a given diagnosis who were transported to HED, followed by the percentage of patients admitted to HED for a given diagnosis made by MET. The last two columns contain information on the number of patients for whom a given MET diagnosis was confirmed by an HED physician. Detailed description of percentage calculations can be found in the legend below the table. Diseases are listed according to the number of patients with a given MET diagnosis who were transferred to HED (i.e., relative to the N_ED_ column). In the table, particular attention should be paid to the fact that when describing the percentages of “verifiability of diagnoses” made by METs (i.e., %_CP_), diagnoses that occurred sporadically may, and even should, be disregarded, e.g., in the table below, it is definitely not worth paying attention to the ICD-10 chapters from the last five lines (one or two patients brought to HED) (Table 6).

A similar comparison was performed for diagnoses categorised into ICD-10 groups. The most common MET diagnoses that resulted in admission to HED are shown in Table 7. The lowest compatibility between MET and HED diagnoses occurred when an emergency physician or the head paramedic did not specify the diagnosis. The highest degree of compatibility was found for injuries and minor diseases, the source of which was not so clearly defined, and was probably related to health deterioration in a chronically ill person (Table 7).

Table 8 presents a summary of medical procedures performed in patients during their stay in HED. The procedures are listed by order of the most common ones. A certain group of medical procedures—such as TRIAGE; emergency nursing care; and NIBP, pulse, and respiration rate measurement—were performed in almost all patients transported to HED (Table 8).

### 3.6. Inpatient Treatment after Admission to Different Departments

Some of the patients transferred to HED received further hospital treatment. This section presents information on which wards the patients were treated, and how long the hospital stay was. The table below shows that slightly less than half of the patients transferred to HED received further in-patient treatment. We presented classification of the wards the patients were referred to for the group of 385 hospitalised patients. The wards are ranked according to the frequency of hospitalisation. Most patients were hospitalised in different cardiology wards, as well as neurology, internal medicine, and pulmonology units. Furthermore, Table 9 presents a detailed distribution of length of stay (LOS) with division into the following periods: 0–4 days, 5–7 days, 8–10 days, 11–14 days, and over 14 days. Most patients stayed in hospital for no longer than 2 weeks (Table 9).

The mean LOS was 10.1 days (median 9.5 days). The minimum LOS was 0 days, which was was reported for three patients who died in the hospital ward immediately after the onset of hospitalisation. The maximum LOS was 46 days, although the majority of patients (over 75%) were hospitalised for no longer than 13 days.

### 3.7. Detailed List of Physician’s Diagnoses for the Selected Diagnoses Made by the Head of MET Based on ICD-10 Groups

For the group “Other forms of heart disease”, diagnostic compatibility was 84.9%. A similarly high rate of diagnostic compatibility was reported for the ICD-10 group “Hypertensive diseases”. It was 68% of all cases in the study group. For the ICD-10 group of diagnoses “Symptoms and signs involving the circulatory and respiratory systems”, the compatibility with HED diagnoses was only 35.4%. A low rate of diagnostic compatibility (24.5%) was also shown for the ICD-10 group “General symptoms and signs”. As for the aforementioned diagnoses of stroke and the consequent brain diseases, the verifiability of the medical diagnosis in the ICD-10 group “Cerebrovascular diseases” was very high (80%). A relatively high diagnostic compatibility between MET (44.6%) and HED was found for the ICD-10 group “symptoms and signs involving the digestive system and abdomen”. The ICD-10 groups of “Injuries to the head” and “Injury to the hip and thigh” showed high diagnostic compatibility between MET and HED. High rates of diagnostic compatibility (73.7%) were also observed in the ICD-10 group “Chronic lower respiratory diseases”. The group of ischaemic heart diseases was also characterised by high agreement with the diagnosis made by an HED doctor on duty.

## 4. Discussion

Determination of the percentage of MET diagnoses confirmed by an HED physician was an important element of our research. Nevertheless, it only made sense for those who were referred by MET heads with the most common diagnoses with which patients were admitted to HED. The diagnostic compatibility was 85% for the ICD-10 group “Other forms of heart disease”. A similarly high diagnostic compatibility was found for the ICD-10 group “Hypertensive diseases” (68%). Diagnostic compatibility between MET and HED for the ICD-10 group “Symptoms and signs involving the circulatory and respiratory systems” was 35.4%. Low diagnostic compatibility (24.5%) was also found for the ICD-10 group “General symptoms and signs”. For strokes and their sequelae, the verifiability of the medical diagnosis in the ICD-10 group “Cerebrovascular diseases” was very high (80%). A high rate of diagnostic concordance (almost 74%) was also confirmed for the ICD-10 groups “Chronic lower respiratory diseases” and “Ischaemic heart diseases”. Christie et al., who analysed the diagnostic compatibility for dyspnoea between METs and HED, found only 67 (22.3%) cases of MET misdiagnosis. Hence, the diagnostic concordance between MET and HED was 77.7%. It was also noted that the diagnostic accuracy for dyspnoea increased with increasing advancement of the MET (three levels of advancement). The highest diagnostic accuracy for dyspnoea was reported for anaphylaxis (100%), followed by asthma-related dyspnoea (86%). Dyspnoea due to pulmonary embolism was the most poorly diagnosed type of dyspnoea (46%) [21]. Williams et al. included 1067 patients identified by paramedics as having respiratory diseases in their research. From all patients transported to HED, 66% were diagnosed by paramedics with asthma, 23% with COPD, whereas others were diagnosed with other upper respiratory diseases (11%). Patients who were transported to HED and underwent broad diagnostic evaluation were diagnosed with asthma (41%), COPD (57%), and respiratory infections classified elsewhere (2%) [22]. In their retrospective analysis of medical records of 810 patients >60 years of age transported to HED due to suspected myocardial ischaemia, Coventery et al. showed 71.4% diagnostic compatibility with HED diagnosis following thorough patient examination [23]. In their study in 495 patients >65 years of age who were transported to HED due to suspected pulmonary oedema caused by acute heart failure, Williams et al. confirmed diagnostic compatibility with HED discharge report for only 37.58% of cases (n = 186) [24]. In the 90s, Schneider et al. conducted a cross-sectional study, which was no less important than current analyses, among patients complaining of chest pain and dyspnoea, and thus presenting with problems with the cardiovascular and respiratory systems. The study included 102 patients with complete medical documentation who were transported by METs to HED. Diagnostic compatibility data were analysed for cardiovascular and respiratory diseases. A statistically significant compatibility was found between paramedic’s diagnosis and the one made by an HED doctor for the circulatory (*p* = 0.0001) and respiratory system (*p* = 0.0001). Generally, the diagnostic concordance between paramedics and HED doctors was 82% (*p* = 0.05) [25]. The verifiability of MET diagnoses with those made by HED doctors is divergent in some analyses. This is due to the fact that METs do not have enough time to help a patient in the event of a life-threatening emergency. In such situations, they must use the “charge and drive” tactic, which in turn leads to a less precise diagnosis. On the other hand, differences in diagnosis occur as a result of excessive stress to which the MET is exposed when dealing with a patient in a life-threatening situation. In this case, the medical personnel use their knowledge to help the patient in the maximum possible way, and making the diagnosis becomes of secondary importance. Furthermore, the diagnostic differences result from the possibility of performing a more extensive diagnosis by HED doctors. Furthermore, an HED has a team of specialists from different departments, who, with access to diagnostic tools and more time at hand, are able to make a correct or more detailed diagnosis. It can also be presumed that the time to reach the correct diagnosis may be longer in older adults. This is due to the fact that geriatric patients have more comorbidities. Therefore, METs need relatively more time to establish a correct diagnosis, and decide whether to transport the patient to HED. It should be noted that a misdiagnosis made by the MET head does not result from a lack of knowledge, skills, or professional experience, as when it comes to neurological, cardiovascular, and respiratory diseases, and the broadly understood “injuries”, the research shows high rates of diagnostic verifiability due to the fact that paramedics and healthcare system doctors are very well-trained in the initial diagnosis and examination in the event of the above-mentioned systemic diseases.

We also analysed medical procedures performed in the HED immediately after patient admission (n = 829). It can be noticed here that some actions on the patient were taken in almost all patients diagnosed in the Emergency Department. Medical segregation (TRIAGE) and emergency nursing care were used in 99.5% of all cases. High percentage values (about 95%) were also reported for such medical procedures, such as blood pressure measurement, pulse, and respiratory rate assessment. Olgers et al. [26] included 270 HED patients in their study. They analysed complete medical segregation, preliminary assessment of vital functions (A—airway; B—breathing; C—circulation; D—disability; E—exposure), as well as steps to stabilise the patient’s condition in the event of a sudden threat to health and life. The study showed that all these measures were feasible for medical personnel in 83% of cases, and were implemented within the first 10 min. A decision not to use the ABCDE approach was often based on the first clinical impression or vital signs during segregation [26]. Dos Santos et al. [27] conducted a cross-sectional study in a general public hospital among 255 older patients > 65 years of age. A visit to HED was defined as an older patient’s stay of ≤24 h. Medical segregation was performed in 99.3% of all patients, whereas the assessment of vital signs (heart rate and blood pressure) was performed in 98.6% of the older individuals. Furthermore, 75.1% of all patients required ECG, intravenous cannula insertion, and pharmacotherapy [27]. The results for medical procedures performed at HED are comparable. This is due to the fact that HEDs, both in Poland and worldwide, have specific procedures at their disposal, and, thus, strive to constantly improve working conditions, while trying to minimise diagnostic errors. Before medical examination by a doctor, lower rank personnel (nurses, paramedics) are obliged to assess and determine the priority of providing help based on medical history, visual assessment of the patient (according to the Glasgow Coma Scale), and, depending on the identified medical problem, are required to measure vital signs: blood pressure, pulse, respiration, glycaemia, oxygen saturation, and to perform ECG. Implementation of other medical procedures depends on the patient’s condition and the decision made by the HED doctor on duty.

Our analysis of hospital stays and treatment in specialist departments of the Provincial Hospitals in Biała Podlaska and Chełm included 385 patients (46.4%) who, after qualification by an HED physician, were hospitalised in different departments. Transport of patients by METs to HEDs in situations other than life and health threats is a problem that was noticed in the United States a long time ago, and concerns most countries in the world. Flores-Mateo et al. showed that 52% of HED visits were non-priority and could be managed by a GP [28]. In their study of 223 HEDs in Poland in 2011, Guła et al. [29] showed that patients in a state of sudden health threat accounted for less than half of all patients hospitalised in one in three HEDs. The percentage of patients requiring further hospital stay did not typically exceed 30% of all reporting patients, whereas the percentage of patients discharged home after HED stay was estimated at 60% [29]. Another example of the overcrowding of HEDs, and overuse of METs was shown by Rzońca and Bednarz, who, based on the analysis of medical records of patients who were transported by METs and referred by GPs (primary health care) to HED in 2013, showed that almost ¾ all patients (72.6%) did not require hospitalisation in specialist hospital wards, and were discharged home after diagnosis and emergency management [30]. A small percentage of hospitalisations after stay at HED among patients referred by their primary care physicians may indicate insufficient access to primary health care services. A statistically significant relationship between the level of primary health care services and the number of hospitalisations was noted in 2008 by Kravet et al. The authors concluded that increasing the rate of primary care physicians reduced the number of HED admissions and visits [31]. In our study, patients were hospitalised in the following departments: cardiology (33.7%), neurology (19.7%), traumatological orthopaedics and surgery (13.8%), internal diseases (8.6%), pulmonology (6.8%), and geriatric (4.9%). As rightly noted by Penson et al., all hospital admissions accounted for 12% to 14% of all HED admissions, of which, 1.3–1.9% were Intensive Care Unit (ICU) admissions, 2.3–4.1% were cardiac unit admissions, 4.3–4.5% were neurology and internal medicine admissions, whereas admissions to traumatology and surgery departments oscillated between 4% and 5.8% [32]. As can be concluded from the above studies, a significant increase in the number of patients transported to HEDs is expected in the coming years, and, thus, the number of patients discharged from HEDs will increase significantly, whereas the number of patients admitted to hospital departments from HEDs will drop. Therefore, it is worth searching for solutions aimed at reducing the number of outpatients in HEDs [33]. The development of competencies and medical services in the field of so-called minor surgery as part of primary health care may be one of solutions. Unfortunately, there is no effective mechanism for limiting the inflow of patients to HEDs, and documenting the refusal of admission to HED is time-consuming and often difficult to rationally justify [33]. Therefore, it is necessary to seek solutions that could encourage GPs to provide medical services on an outpatient basis. One such incentive may be a significant financial injection directed at primary health centres, which would significantly relieve Emergency Medical Services, and, thus, only patients in a state of sudden health threat would be brought to HEDs.

Our study has some limitations. First of all, it covered medical documentation from only two exemplary medical emergency service stations in cities with a county status, to which we had the easiest access. Therefore, the study needs to be extended in order to be representative of the entire population of older adults in Poland. Secondly, the analysed group of patients is characterised by an over-representation of women in relation to men. A larger (comparable) number of men should be included in future studies.

## 5. Conclusions

Differences between the initial diagnosis made by the heads of the METs and the diagnosis made by the doctor on duty in the HED depended on the chapter of diseases in the ICD-10 classification, but they were acceptable. The majority of the patients (almost three-quarters) were transported by METs to HEDs. The most common groups of diseases that require HED admission include cardiovascular diseases, injuries due to external causes, and respiratory diseases. A moderate percentage of patients transferred to HEDs were qualified for further specialist treatment in hospital departments.

Our research showed that more than 50% of Hospital Emergency Department admissions were potentially avoidable. A possible solution to this problem is to identify groups of older patients characterised by a higher risk of being admitted to Hospital Emergency Departments. This process could help develop a program of interventions that fill the national healthcare system gaps at the community level, and prevent older people from resorting to Hospital Emergency Department admissions which are economically inefficient for the healthcare system.

## Figures and Tables

**Table 1:** Socio-demographic characteristics of patients included in the analysis.

Variable		n	%
sex	female	702	58.5
	male	498	41.5
place of residence	urban	592	49.3
	rural	608	50.7
education	elementary	80	6.7
	vocational	589	49.1
	secondary	311	25.9
	higher	220	18.3
marital status	married	566	47.2
	single	79	6.6
	divorced	50	4.2
	widowed	505	42.1

**Table 2:** MET head diagnosis by ICD-10 classification.

Diagnosis Made by the MET Head—ICD-10 Chapters	n	% ^(1)^
IX. Cardiovascular diseases	482	40.2
XVIII. Clinical and laboratory findings, not elsewhere classified	452	37.7
XIX. Injury, poisoning, and certain other consequences of external causes	151	12.6
X. Diseases of the respiratory system	56	4.7
IV. Endocrine, nutritional, and metabolic diseases	41	3.4
V. Mental and behavioural disorders	31	2.6
II. Cancer	24	2
VI. Diseases of the nervous system	23	1.9
XIV. Diseases of the genitourinary system	22	1.8
XX. External causes of morbidity and mortality	18	1.5
XXI. Factors influencing health status and contact with health services	16	1.3
XI. Diseases of the digestive system	12	1
XIII. Diseases of the musculoskeletal system and connective tissue	8	0.7
I. Certain infectious and parasitic diseases	2	0.2
III. Diseases of the blood and blood-forming organs, and certain disorders involving the immune mechanism	2	0.2
XII. Diseases of the skin and subcutaneous tissue	2	0.2

^(1)^ The sum does not have to be 100%, as more than one diagnosis may have been made in one patient.

**Table 3:** The percentage distribution of the frequency of the most common disease groups diagnosed by the MET head.

Diagnosis Made by the MET Head		n	% ^(1)^	% ^(2)^
Chapter IX diseases	Other forms of heart disease	241	20.1	47.5
	Hypertension	154	12.8	30.4
	Cerebrovascular diseases	65	5.4	12.8
	Ischaemic heart disease	28	2.3	5.5
	Diseases of arteries, arterioles, and capillaries	11	0.9	2.2
	Diseases of veins, lymphatic vessels, and lymph nodes, not elsewhere classified	4	0.3	0.8
	Other and unspecified disorders of the circulatory system	4	0.3	0.8
Chapter XVIII diseases	General symptoms and signs	184	15.3	37.8
	Symptoms and signs involving the circulatory and respiratory systems	146	12.2	30
	Symptoms and signs involving the digestive system and abdomen	91	7.6	18.7
	Ill-defined and unknown causes of mortality	36	3	7.4
	Symptoms and signs involving cognition, perception, emotional state	12	1	2.5
	Symptoms and signs involving the genitourinary system	11	0.9	2.3
	Abnormal findings on examination of blood, without diagnosis	4	0.3	0.8
	Symptoms and signs involving the skin and subcutaneous tissue	1	0.1	0.2
	Symptoms and signs involving the nervous and musculoskeletal systems	1	0.1	0.2
	Symptoms and signs involving speech and voice	1	0.1	0.2
Chapter XIX diseases	Injuries to the head	51	4.3	31.9
	Injuries to the hip and thigh	39	3.3	24.4
	Injuries to the shoulder and upper arm	14	1.2	8.8
	Injuries to the knee and lower leg	13	1.1	8.1
	Injuries to the thorax	12	1	7.5
	Injuries to the ankle and foot	9	0.8	5.6
	Injuries to the wrist, hand, and fingers	6	0.5	3.8
	Other and unspecified effects of external causes	4	0.3	2.5
	Injuries to the abdomen, lower back, lumbar spine, and pelvis	3	0.3	1.9
	Toxic effects of substances chiefly nonmedicinal as to source	3	0.3	1.9
	Injuries to the neck	2	0.2	1.3
	Injuries to the elbow and forearm	2	0.2	1.3
	Injuries involving multiple body regions	2	0.2	1.3
Chapter X diseases	Chronic lower respiratory diseases	35	2.9	62.5
	Influenza and pneumonia	7	0.6	12.5
	Other diseases of the respiratory system	5	0.4	8.9
	Acute upper respiratory infections	4	0.3	7.1
	Other respiratory diseases principally affecting the interstitium	3	0.3	5.4
	Other acute lower respiratory infections	1	0.1	1.8
	Other diseases of the pleura	1	0.1	1.8
Chapter IV diseases	Diabetes mellitus	31	2.6	75.6
	Other disorders of glucose regulation and pancreatic internal secretion	6	0.5	14.6
	Metabolic disorders	4	0.3	9.8
Chapter V diseases	Neurotic, stress-related, and somatoform disorders	10	0.8	32.3
	Organic or symptomatic mental disorders	7	0.6	22.6
	Mental and behavioural disorders due to psychoactive substance use	7	0.6	22.6
	Mood [affective] disorders	2	0.2	6.5
	Unspecified mental disorders	2	0.2	6.5
	Schizophrenia, schizotypal, delusional, and other non-mood psychotic disorders	1	0.1	3.2
	Disorders of adult personality and behaviour	1	0.1	3.2
	Behavioural and emotional disorders with onset usually occurring in childhood and adolescence	1	0.1	3.2

^(1)^ percentage of patients with a given group of diseases; ^(2)^ percentage of diseases in given chapter.

**Table 4:** The most common diagnoses made by an HED doctor on duty.

Chapter (HED)	n	%
IX. Cardiovascular diseases	372	44.9
XIX. Injury, poisoning, and certain other consequences of external causes	132	15.9
XVIII. Signs and abnormal clinical and laboratory findings, not elsewhere classified	131	15.8
X. Diseases of the respiratory system	56	6.8
VI. Diseases of the nervous system	30	3.6
XI. Diseases of the digestive system	19	2.3
IV. Endocrine, nutritional, and metabolic diseases	17	2.1
XIV. Diseases of the genitourinary system	16	1.9
II. Cancer	14	1.7
V. Mental and behavioural disorders	12	1.4
I. Certain infectious and parasitic diseases	7	0.8
XIII. Diseases of the musculoskeletal system and connective tissue	7	0.8
XX. External causes of morbidity and mortality	6	0.7
VIII. Diseases of the ear and mastoid process	5	0.6
III. Diseases of the blood and blood-forming organs, and certain disorders involving the immune mechanism	3	0.4
XXI. Factors influencing health status and contact with health services	2	0.2

**Table 5:** Percentage distribution of the most common disease groups diagnosed by an HED doctor.

Diagnosis Made by an HED Doctor		n	% ^(1)^	% ^(2)^
Chapter IX diseases	Other forms of heart disease	199	24	53.5
	Cerebrovascular diseases	59	7.1	15.9
	Hypertension	54	6.5	14.5
	Ischaemic heart disease	47	5.7	12.6
	Diseases of veins, lymphatic vessels, and lymph nodes, not elsewhere classified	9	1.1	2.4
	Diseases of arteries, arterioles, and capillaries	4	0.5	1.1
Chapter XIX diseases	Injuries to the head	43	5.2	32.6
	Injuries to the hip and thigh	35	4.2	26.5
	Injuries to the shoulder and upper arm	13	1.6	9.8
	Injuries to the knee and lower leg	9	1.1	6.8
	Injuries to the thorax	8	1	6.1
	Injuries to the ankle and foot	7	0.8	5.3
	Toxic effects of substances chiefly nonmedicinal as to source	3	0.4	2.3
	Other and unspecified effects of external causes	3	0.4	2.3
	Injuries to the neck	2	0.2	1.5
	Injuries to the abdomen, lower back, lumbar spine, and pelvis	2	0.2	1.5
	Injuries to the elbow and forearm	2	0.2	1.5
	Injuries to the wrist, hand, and fingers	2	0.2	1.5
	Injuries involving multiple body regions	2	0.2	1.5
	Complications of surgical and medical care, not elsewhere classified	1	0.1	0.8
Chapter XVIII diseases	Symptoms and signs involving the circulatory and respiratory systems	55	6.6	42
	General symptoms and signs	34	4.1	26
	Symptoms and signs involving the digestive system and abdomen	29	3.5	22.1
	Symptoms and signs involving the genitourinary system	9	1.1	6.9
	Abnormal findings on examination of blood, without diagnosis	4	0.5	3.1
Chapter X diseases	Chronic lower respiratory diseases	19	2.3	33.9
	Influenza and pneumonia	18	2.2	32.1
	Other diseases of the respiratory system	6	0.7	10.7
	Other respiratory diseases principally affecting the interstitium	5	0.6	8.9
	Other acute lower respiratory infections	4	0.5	7.1
	Other diseases of the pleura	2	0.2	3.6
	Acute upper respiratory infections	1	0.1	1.8
	Lung diseases due to external agents	1	0.1	1.8
Chapter VI diseases	Episodic and paroxysmal disorders	25	3	83.3
	Other disorders of nervous system	3	0.4	10
	Nerve, nerve root, and plexus disorders	2	0.2	6.7

^(1)^ percentage of patients with a given group of diseases; ^(2)^ percentage of diseases in given chapter.

**Table 6:** Percentage of patients transported to HED by MET diagnosis according to ICD-10 Chapters.

ICD-10 Chapters	N_MET_	N_HED_	%_HED_	N_CP_	%_CP_
IX. Cardiovascular diseases	482	368	76.3	314	85.3
XVIII. Clinical and laboratory findings, not elsewhere classified	452	292	64.6	118	40.4
XIX. Injury, poisoning, and certain other consequences of external causes	151	133	88.1	122	91.7
X. Diseases of the respiratory system	56	35	62.5	30	85.7
IV. Endocrine, nutritional, and metabolic diseases	41	23	56.1	9	39.1
VI. Diseases of the nervous system	23	17	73.9	10	58.8
XIV. Diseases of the genitourinary system	22	16	72.7	13	81.3
II. Cancer	24	13	54.2	9	69.2
V. Mental and behavioural disorders	31	12	38.7	8	66.7
XX. External causes of morbidity and mortality	18	12	66.7	2	16.7
XI. Diseases of the digestive system	12	10	83.3	9	90
XXI. External causes of morbidity and mortality	16	2	12.5	0	0
XIII. Diseases of the musculoskeletal system and connective tissue	8	2	25	2	100
I. Certain infectious and parasitic diseases	2	2	100	1	50
III. Diseases of the blood and blood-forming organs, and certain disorders involving the immune mechanism	2	2	100	0	0
XII. Diseases of the skin and subcutaneous tissue	2	1	50	0	0

N_MET_—number of patients with a given MET diagnosis; N_HED_—number of patients with a given diagnosis, who were transported to HED; %_HED_—percentage of patients with a given diagnosis, who were transported to HED (N_HED_/N_MET_); N_CP_—number of patients with a given MET diagnosis confirmed by HED doctor; %_CP_—percentage of patients with a given MET diagnosis confirmed by HED doctor (N_CP_/N_HED_).

**Table 7:** The most common groups of (ICD-10) MET diagnoses leading to ED admission.

ICD-10 Groups	N_MET_	N_HED_	%_HED_	N_CP_	%_CP_
Other forms of heart disease	241	205	85.1	174	84.9
Symptoms and signs involving the circulatory and respiratory systems	146	130	89	46	35.4
General symptoms and signs	184	110	59.8	27	24.5
Hypertension	154	75	48.7	51	68
Cerebrovascular diseases	65	65	100	52	80
Symptoms and signs involving the digestive system and abdomen	91	56	61.5	25	44.6
Injuries to the head	51	43	84.3	39	90.7
Injuries to the hip and thigh	39	37	94.9	32	86.5
Ischaemic heart disease	28	27	96.4	19	70.4
Chronic lower respiratory diseases	36	19	52.8	14	73.7
Diabetes mellitus	31	16	51.6	3	18.8
Episodic and paroxysmal disorders	19	14	73.7	10	71.4
Injuries to the shoulder and upper arm	14	14	100	11	78.6
Injuries to the knee and lower leg	13	11	84.6	7	63.6
Injuries to the thorax	12	10	83.3	8	80

**Table 8:** Emergency procedures performed in HED.

Patient Management in HED	n	% ^(1)^
TRIAGE	825	99.5
Emergency nursing care	825	99.5
NIBP	814	98.2
Pulse	794	95.8
Breath	776	93.6
IV Cannula	693	83.6
ECG	632	76.2
Blood for testing	612	73.8
Pharmacotherapy	572	69
Specialist consultation	538	64.9
X-ray	318	38.4
Cardiac panel	253	30.5
SpO_2_	244	29.4
CT	194	23.4
Glucose	184	22.2
Oxygen	153	18.5
Temperature	142	17.1
Stroke panel	104	12.5
Ultrasound	95	11.5
Cardioversion	68	8.2
Dressing	61	7.4
Wound suturing	44	5.3
Plaster immobilisation	36	4.3
Urine test/catheter	35	4.2
Intubation	16	1.9
Defibrillation	6	0.7
No data/no actions	4	0.5

^(1)^ The sum does not have to be 100%, as it was possible to indicate any number of answer variants.

**Table 9:** In-patient treatment of patients admitted to different departments.

Variable		n	%
HED doctor’s decision	Discharge home	444	53.6
	In-hospital treatment	385	46.4
Department	Neurology	76	19.7
	Cardiology	72	18.7
	Conservative cardiology	41	10.6
	Internal Medicine	33	8.6
	Pulmonology	26	6.8
	Traumatology and orthopaedics with spine surgery	22	5.7
	Geriatrics	19	4.9
	Surgery	18	4.7
	Invasive cardiology	17	4.4
	Traumatology and orthopaedics	13	3.4
	Intensive care	8	2.1
	Urology	8	2.1
	Anaesthesiology and intensive therapy	7	1.8
	Department of observation and infectious diseases	6	1.6
	Oncology	6	1.6
	Psychiatric	5	1.3
	Gynaecology and obstetrics	4	1
	ENT with the ophthalmology subdepartment	1	0.3
	Otolaryngology	1	0.3
	Palliative	1	0.3
	Nursing care	1	0.3
Length of stay at a given Department [days]	0–4	34	8.9
	5–7	85	22.1
	8–10	99	25.8
	11–14	111	28.9
	≥15	55	14.3

## Data Availability

Data are available upon reasonable request.

## References

[1] United Nations (2020). World Population Ageing 2020: Highlights.

[2] United Nations (2017). World Population Ageing 2017: Highlights.

[3] Statistics Poland (2014). Demographic Situation of the Elderly and the Consequences of the Aging of the Polish Population in the Light of the Forecast for 2014–2050.

[4] Beard J.R., Officer A., de Carvalho I.A., Sadana R., Pot A.M., Michel J.P., Lloyd-Sherlock P., Epping-Jordan J.E., Peeters G.M.E.E.G., Mahanani W.R. (2016). The World report on ageing and health: A policy framework for healthy ageing. Lancet.

[5] Wajnberg A., Hwang U., Torres L., Yang S. (2012). Characteristics of frequent geriatric users of an urban emergency department. J. Emerg. Med..

[6] Rehn M., Sollid S.J. (2013). Letter to the editor. Air Med. J..

[7] Sinha S.K., Bessman E.S., Flomenbaum N., Leff B. (2011). A systematic review and qualitative analysis to inform the development of a new emergency department-based geriatric case management model. Ann. Emerg. Med..

[8] Aminzadeh F., Dalziel W.B. (2002). Older adults in the emergency department: A systematic review of patterns of use, adverse outcomes, and effectiveness of interventions. Ann. Emerg. Med..

[9] Keim S., Sanders A., Meldon S., Ma O.J., Woolard R. (2004). Geriatric emergency department use and care. Geriatric Emergency Medicine.

[10] Sanders A.B., Morley J.E. (1993). The older person and the emergency department. J. Am. Geriatr. Soc..

[11] Reher D., Requena M. (2018). Living Alone in Later Life: A Global Perspective. Popul. Dev. Rev..

[12] Fisher K.A., Griffith L.E., Gruneir A., Upshur R., Perez R., Favotto L., Nguyen F., Markle-Reid M., Ploeg J. (2021). Effect of socio-demographic and health factors on the association between multimorbidity and acute care service use: Population-based survey linked to health administrative data. BMC Health Serv. Res..

[13] Agosti P., Tettamanti M., Vella F.S., Suppressa P., Pasina L., Franchi C., Nobili A., Mannucci P.M., Sabbà C., Reposi Investigators (2018). Living alone as an independent predictor of prolonged length of hospital stay and non-home discharge in older patients. Eur. J. Intern. Med..

[14] Carpar E., McCarthy G., Adamis D., Donmezler G., Cesur E., Fistikci N. (2018). Socio-demographic characteristics and factors associated with hospitalization in psychiatry of old age patients: An international comparison between Ireland and Turkey. Aging Clin. Exp. Res..

[15] Barrenetxea J., Tan K.B., Tong R., Chua K., Feng Q., Koh W.P., Chen C. (2021). Emergency hospital admissions among older adults living alone in the community. BMC Health Serv. Res..

[16] Hughes J.M., Freiermuth C.E., Shepherd-Banigan M., Ragsdale L., Eucker S.A., Goldstein K., Hastings S.N., Rodriguez R.L., Fulton J., Ramos K. (2019). Emergency Department Interventions for Older Adults: A Systematic Review. J. Am. Geriatr. Soc..

[17] Carpenter C.R., Platts-Mills T.F. (2013). Evolving prehospital, emergency department, and “inpatient” management models for geriatric emergencies. Clin. Geriatr. Med..

[18] Nunes B.P., Soares M.U., Wachs L.S., Volz P.M., Saes M.O., Duro S.M.S., Thumé E., Facchini L.A. (2017). Hospitalization in older adults: Association with multimorbidity, primary health care and private health plan. Rev. Saude Publica.

[19] Dutra M.M., Moriguchi E.H., Lampert M.A., Poli-de-Figueiredo C.E. (2011). Predictive validity of a questionnaire to identify older adults at risk for hospitalization. Rev. Saude Publica.

[20] Rozporządzenie Ministra Zdrowia z Dnia 9 Listopada 2015 r. w Sprawie Rodzajów, Zakresu i Wzorów Dokumentacji Medycznej Oraz Sposobu jej Przetwarzania (Dz.U. 2015, poz. 2069). https://isap.sejm.gov.pl/isap.nsf/download.xsp/WDU20150002069/O/D20152069.pdf.

[21] Christie A., Costa-Scorse B., Nicholls M., Jones P., Howie G. (2016). Accuracy of working diagnosis by paramedics for patients presenting with dyspnoea. Emerg. Med. Australas..

[22] Williams T.A., Finn J., Fatovich D., Perkins G.D., Summers Q., Jacobs I. (2015). Paramedic Differentiation of Asthma and COPD in the Prehospital Setting Is Difficult. Prehosp. Emerg. Care.

[23] Coventry L.L., Bremner A.P., Williams T.A., Jacobs I.G., Finn J. (2014). Symptoms of myocardial infarction: Concordance between paramedic and hospital records. Prehosp. Emerg. Care.

[24] Williams T.A., Finn J., Celenza A., Teng T.H., Jacobs I.G. (2013). Paramedic identification of acute pulmonary edema in a metropolitan ambulance service. Prehosp. Emerg. Care.

[25] Schaider J.J., Riccio J.C., Rydman R.J., Pons P.T. (1995). Paramedic diagnostic accuracy for patients complaining of chest pain or shortness of breath. Prehosp. Disaster Med..

[26] Olgers T.J., Dijkstra R.S., Drost-de Klerck A.M., Ter Maaten J.C. (2017). The ABCDE primary assessment in the emergency department in medically ill patients: An observational pilot study. Neth. J. Med..

[27] Santos F.S.D., Dias B.M., Reis A.M.M. (2019). Emergency department visits of older adults within 30 days of discharge: Analysis from the pharmacotherapy perspective. Einstein.

[28] Flores-Mateo G., Violan-Fors C., Carrillo-Santisteve P., Peiró S., Argimon J.M. (2012). Effectiveness of organizational interventions to reduce emergency department utilization: A systematic review. PLoS ONE.

[29] Guła P., Kutaj-Wąsikowska H., Kalinowski M. (2012). A model of emergency department throughput in Poland. J. Orthop. Trauma Surg. Rel. Res..

[30] Rzońca P., Bednarz K. (2013). The role of the Basic Health Care doctor in helping injured patients. Analysis of medical records of an Emergency Room. Fam. Med. Prim. Care Rev..

[31] Kravet S.J., Shore A.D., Miller R., Green G.B., Kolodner K., Wright S.M. (2008). Health care utilization and the proportion of primary care physicians. Am. J. Med..

[32] Penson R., Coleman P., Mason S., Nicholl J. (2012). Why do patients with minor or moderate conditions that could be managed in other settings attend the emergency department?. Emerg. Med. J..

[33] Guła P., Karwan K. (2012). Lean analysis in the ED operating procedures, based on the author’s experience. Lek. Wojsk..

